# Bacteria, Phages and Septicemia

**DOI:** 10.1371/journal.pone.0001145

**Published:** 2007-11-07

**Authors:** Aušra Gaidelytė, Martti Vaara, Dennis H. Bamford

**Affiliations:** 1 Department of Biological and Environmental Sciences, Institute of Biotechnology, University of Helsinki, Helsinki, Finland; 2 Department of Clinical Microbiology, Helsinki University Hospital, Helsinki, Finland; The Scripps Research Institute, United States of America

## Abstract

The use of phages is an attractive option to battle antibiotic resistant bacteria in certain bacterial infections, but the role of phage ecology in bacterial infections is obscure. Here we surveyed the phage ecology in septicemia, the most severe type of bacterial infection. We observed that the majority of the bacterial isolates from septicemia patients spontaneously secreted phages active against other isolates of the same bacterial strain, but not to the strain causing the disease. Such phages were also detected in the initial blood cultures, indicating that phages are circulating in the blood at the onset of sepsis. The fact that most of the septicemic bacterial isolates carry functional prophages suggests an active role of phages in bacterial infections. Apparently, prophages present in sepsis-causing bacterial clones play a role in clonal selection during bacterial invasion.

## Introduction

Septicemia is a serious medical condition where bacteria present in the blood circulatory system provoke an amplified and dysregulated immune response in the individual. The most common infection sites leading to bacterial entry into the circulatory system are bacterial infections in the lungs, urinary tract, abdominal cavity, and primary infections of the bloodstream [1]. Rapid antibiotic intervention is currently the only way to treat septicemia (as well as other bacterial infections). However, many bacterial pathogens have become resistant to antibiotic regimens, resulting in an urgent health problem worldwide [2], [3]. One potentially useful method for the treatment of antibiotic resistant bacterial infections employs bacterial viruses called bacteriophages (also known as phages) capable of killing bacteria [4]–[7]. They were widely used to treat bacterial infections since their discovery in the beginning of the twentieth century, but their use was neglected in western countries after the discovery of antibiotics [6], [8]. The modern application of phages in parts of the world that require documented and scientifically controlled clinical experiments is limited to the protection of ready-to-eat meat and poultry products [9]. Phage derived enzymes lytic to Gram-positive bacteria are the most promising candidates to enter the markets for therapeutic use [10], [11].

In contrast to virulent phages, which kill bacteria immediately, temperate phages integrate their genomes into bacterial chromosomes to establish a prophage state. Prophages and other genetic elements such as transposons, plasmids, and pathogenicity islands encode virulence factors [12]. Temperate phages disseminate virulence genes and thus contribute to the evolution and emergence of new pathogenic bacteria. Only the most virulent bacterial clones are capable of tissue invasion possibly leading to septicemia, as the bacteria have to overcome anatomical and host immune system barriers to enter the circulatory system. Although there is considerable information regarding prophages [13], [14] and phage-encoded virulence factors in bacterial pathogens [15], few studies have investigated microbial ecology in clinical bacterial infections. Here we surveyed the phage ecology in septicemia, the most severe type of bacterial infection. We observed that the majority of septicemia-causing bacteria could be induced to produce phages active against other isolates of the same bacterial strain. Such phages were also detected in the initial blood cultures, indicating that phages are circulating in the blood at the onset of sepsis. Further characterization of the phage isolates revealed that the virus detected in the blood culture was the same as induced from the bacterium isolated from that particular blood culture sample.

## Results

### The Majority of Septicemia-Causing Bacteria Can Be Induced to Produce Phages

In this report we investigated *Escherichia coli* (Ec), *Pseudomonas aeruginosa* (Pa), *Staphylococcus aureus* (Sa) and *Klebsiella pneumoniae* (Kp) isolates from septicemia patients. Two sets of bacterial isolates and blood culture samples were obtained (Set I 150 and Set II 30 bacterial isolates, Table S1). Set I samples were analyzed by plating the blood culture sample (stored at –80°C) with the homologous bacterial strain. (Homologous strain refers to the strain originally isolated from that particular blood culture sample. Heterologous strains are other bacterial strains isolated from blood culture samples or elsewhere.) None of the blood culture samples contained plaque-producing viruses, indicating that no virulent phages were present. However, eight Pa strains produced plaques when plated without the blood culture sample (10^2^–10^4^ PFU/ml) (Table S1). These strains were likely lysogens that spontaneously released viruses and were sensitive to their own viruses. This result suggested that prophages resided in the bacterial isolates. Ten randomly selected heterologous strains from Set I and elsewhere were then used as indicators to test for spontaneous phage induction. Prophages were also induced with UV and mitomycin C (MitC) under conditions that reduced the host cell viability by several orders of magnitude. Mid-logarithmic cell culture supernatants were then probed for phage induction by plaque assay. Phages were detected in 91 out of 150 culture supernatants (Table 1) ranging from 10 to 10^7^ PFU/ml. Plaques appeared only on one indicator strain in about half of the cases, suggesting that a larger indicator set could result in the detection of more phage-producing strains. Three virus-producing clones were tested for the type of phage or phages released for all four bacterial strains using the sensitivity pattern of the indicator strains. Only a single pattern was detected in most of the test cases. These data indicate that the bacterial clones released one or only a few types of phages.

**Table 1:** Number of Set I bacterial isolates producing spontaneous or induced plaques.

Bacteria	Spontaneous	MitC	UV	Total unique /all isolates
*E. coli*	25	40	45	57/90
*S. aureus*	7	13	8	16/26
*P. aeruginosa*	16	16	15	16/16
*K. pneumoniae*	2	2	2	2/18
			**Total:**	**91/150**

We next tested if antibiotics commonly used to treat septicemic infections could induce plaque formation in clinical bacterial isolates. We selected five strains (3 Ec and 2 Sa) that did not produce plaques spontaneously but did so after treatment with MitC or UV, to test this idea. Indicator strains (one indicator strain per bacterial isolate) were selected based on data from the experiment described above. We did not observe plaque formation when the cells were treated with tobramycin. However, ciprofloxacin induced phage release from one of the Sa strains. It has been shown previously that ciprofloxacin treatment causes prophage induction (and virulence modulation) in *S. aureus* from patients with cystic fibrosis [16] as well as in shiga-toxin producing *E. coli* strains of human origin [17]–[19]. In some cases, like infections with enterohemorrhagic *E. coli* O157:H7, antibiotic treatment is controversial because of prophage induction, increasing the risk of the hemolytic-uremic syndrome [18], [20].

### Selected Phage-Sensitive Septicemia-Causing Hosts Allowed to Detect High Virus Titers in Original Blood Culture Samples

Results described above indicate that most (if not all) of the bacteria studied here are lysogenic, suggesting that there should be phages present in the original blood culture samples due to spontaneous or induced induction. Using the indicator bacterial strains identified in the lysogeny screening, blood culture samples from Set I (Table S1) were subjected to plaque assay. Plaques were detected in 10 of the 149 samples (∼7%, Table 2). This assay was performed several months after the blood collection and the samples had been frozen and thawed. Freezing has an adverse effect on the virus viability. To eliminate this discrepancy we collected another set of samples (Set II, n = 30) and screened them for possible plaque-forming phages two days after sample collection (Table S1). We also increased the number of possible indicator strains used in the analyses. We observed plaque formation in 10 of the 30 (33%) blood culture samples, with titers ranging from 10 to 10^6^ PFU/ml (Table 3). In most cases when phages were detected in the blood culture samples the corresponding bacterial isolate spontaneously released phages capable of infecting the same indicator strain (Table S1).

**Table 2:** Phage titer of Set I blood culture samples detected on selected indicator strains.

Blood culture sample (Set I)	Indicator strain	Titer (PFU/ml)
***E. coli*** ** (6/90) ∼7%**		
05vv1387	Ec1457	2×10^4^
05vv1522	YMC	1×10^5^
05vv1558	Ec1522	2×10^3^
05vv1809	YMC	2×10^2^
05vv1999	Ec2424	1×10^2^
05vv2388	Ec1643	2×10^3^
***S. aureus*** ** (0/26) 0%**		
***P. aeruginosa*** ** (3/16) ∼19%**		
05vv1315	Pa1400	6×10^3^
05vv1400	Pa1414	2×10^3^
05vv1973	Pa1651	5×10^4^
***K. pneumoniae*** ** (1/18) ∼6%**		
05vv2343	Kp1752	1×10^4^

**Table 3:** Phage titer of Set II blood culture samples detected on indicator strains with the best efficiency of plating.

Blood culture sample (Set II)	Indicator strain	Titer (PFU/ml)
***E. coli*** ** (4/14) ∼29%**		
06vv2974n	Ec1457	∼2×10^3^
06vv2987a	YMC	2×10^4^
06vv3183n	Ec1522	1.4×10^6^
06vv3242	Ec1522	1×10^5^
***S. aureus*** ** (5/12) ∼42%**		
06vv2986a	Sa1912	∼4×10^3^
06vv3106a	Sa1912	10^1^
06vv3133n	Sa1912	4×10^1^
06vv3189n	Sa1912	3×10^5^
06vv3244	Sa1263	1×10^3^
***P. aeruginosa*** ** (0/3) 0%**		
***K. pneumoniae*** ** (1/1) 100%**		
06VT145a	Kp1752	2×10^3^

### The Virus Induced from a Septicemic Bacterium Is Always the Same as Detected in the Original Blood Culture Sample

**Figure 1:**
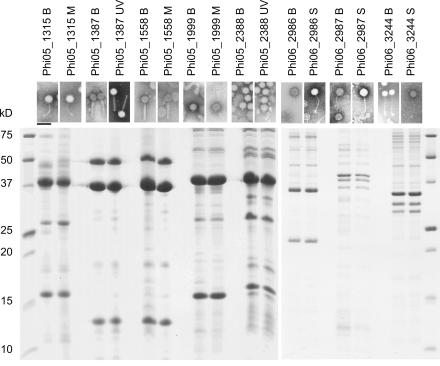
Characterization of phage isolates grown to high titers. Upper panel, electron micrographs of negatively stained phage preparations. Bar, 100 nm. Lower panel, SDS-PAGE analysis of the same phage preparations. The first and last lanes of the gel contain protein size standards (BioRad). Molecular weights of protein standards are indicated on the left.

Attempts to grow the isolated phages to high titer and to purify them using polyethylene glycol precipitation and rate zonal sucrose gradient centrifugation succeeded in 36 cases (24 from Set I and 12 from Set II, Table S2). Phage morphology was determined by negative staining electron microscopy, and the virion structural protein pattern was determined by electrophoresis (SDS-PAGE). Thirty-four of the phages showed a typical tailed dsDNA phage morphology characteristic of myo-, sipho-, or podoviruses, with a head diameter often about 50 nm. Two of the phage isolates were filamentous. We did not detect any other morphotypes. For eleven of the samples both the blood culture phage isolate and the phage isolate induced from homologous lysogenic bacterium were recovered (designated as a phage pair). Interestingly, the pairs were always identical when the morphologies and structural protein patterns of these phage pairs were analyzed (Figure 1). We also detected identical (based on structural protein patterns) phage isolates (not pairs) from the blood culture samples or induced from lysogenic bacteria. The highest number (seven) of identical isolates was detected from Ec. All these isolates infected the same bacterial strain (YMC). Likewise, the two filamentous Pa phages had the same structural protein pattern and morphology. These data indicated that common phage strains were circulating in this hospital setting.

## Discussion

Many bacterial genomes carry both defective and functional prophages [13], [14]. These greatly contribute to the bacterial phenotype and can alter important biological properties.

Bacterial cells carrying prophages usually are immune to the infection of the same phage type. Interestingly, we observed that eight auto-induced Pa phages and two staphylococcal phages (Phi05_1554 and Phi05_1912a) infected their homologous hosts. When several potential heterologous hosts (of the same bacterial strain) were used, a spontaneous or induced phage production was detected in 95 out of 180 isolates. Since more phages were detected when the indicator test set was increased, it is possible that the observed inducible phages (and lysogenic bacteria) represent only a minimal estimate of the actual total. Similarly, the number of phages detected in the blood culture samples is also likely a minimal estimate because of virus decay and possible suboptimal indicator host usage. The observation that the induced phages were consistently identical to those found in the homologous blood culture sample indicates that there are both bacteria and phages circulating in the blood of septicemic patients. The fact that most of the sepsis-causing bacteria carry functional prophages suggests an active role of phages in bacterial infections. Phages released from majority of septicemic bacterial isolates are active against other isolates of the same bacterial strain, but not to the strain causing the disease. Apparently, prophages present in sepsis-causing bacterial clones play a role in clonal selection during bacterial invasion. A similar concept has been proposed for *Streptococcus pneumoniae* isolated from the nasopharynx [21]. Prophage MM1 improves bacterial adherence to the surface of pharyngeal cells and thus contributes to the pathogen fitness and its persistence in humans. It is also likely that bacteriocins are induced from sepsis-causing bacteria. Our preliminary studies on the filamentous Pa phages isolated here show that large amounts of pyocins (Pa bacteriocins) are released upon phage production. Obviously a complex ecosystem is generated in septicemia where the human immune system and bacteria, phages and possibly bacteriocins operate simultaneously with fatal consequences to the infected individual.

The observations made here support the idea that viruses are found in practically all ecological niches, including bacterial infections. Therefore, the therapeutic use of phages in septicemic patients relies on the understanding of the ecosystem created by the patient, pathogenic bacteria, phages, and possible bacteriocins.

## Materials and Methods

### Study design

The samples used in this study consisted of *E. coli*, *K. pneumoniae*, *S. aureus* and *P. aeruginosa* strains originating from septicemic patients isolated from consecutive blood cultures taken from those patients. They also consisted of fluid samples taken from the corresponding blood cultures. Only one sample per patient was included to exclude duplicates. All samples are listed in Table S1. Personal data were removed from all material related to the study to protect the anonymity of the patients. According to ethical principles stated in the Declaration of Helsinki this survey did not involve identifiable human material or identifiable patient data and thus ethical approval was not deemed necessary. Isolates and blood culture samples were collected in the Helsinki University Hospital laboratory during two time periods (April 27 through August 5, 2005 and August 29 through September 25, 2006). During the first time period, 149 blood culture samples and 150 bacterial strains were collected and frozen at –80°C until use (Set I). During the second time period 30 blood culture samples and 30 bacterial isolates were obtained and processed immediately (Set II). Three different specimens with the same origin were marked with the same four-digit number: blood sample (05vv or 06vv), bacterial isolate (Ec for *E. coli*, Sa for *S. aureus*, Pa for *P. aeruginosa*, or Kp for *K. pneumoniae*), and phage (Phi). Common laboratory strains including *E. coli* K12 YMC, *P. aeruginosa* K, O5(R18), O(pJB10), O1(pLM2), and O were also used. Bacteria were cultivated in Luria-Bertani broth (LB) at 37°C with aeration. Cell density was measured using a Klett colorimeter (A_540_) manufactured by Bel-Art Products.

### Phage quantitation

To quantitate virulent phages in blood culture samples, the sample was cleared by centrifugation for five min at 13,000 rpm at 22°C (Heraeus Biofuge) and plated with a suspension of the bacterium originally isolated from that sample (homologous bacterium). To quantitate temperate phages, cleared blood culture samples were diluted up to 100-fold and plated with selected indicator strains (see below). To detect spontaneous phages in culture supernatants of bacteria originally isolated from blood culture samples, bacterial isolates were grown to 200 Klett units at 37°C with aeration, sedimented by centrifugation for five min at 13,000 rpm at 22°C (Heraeus Biofuge), and serial 10-fold dilutions of supernatants were plated with selected indicator strains.

### Selected potential indicator strains for Set I

For *E. coli*, Ec1255, Ec1457, Ec1522, Ec1643, Ec1685, Ec1758, Ec1809, Ec1910, Ec2311, Ec2424, and YMC were used as potential indicator strains. For *S. aureus*, Sa1252, Sa1303, Sa1433, Sa1469, Sa1582, Sa1631, Sa1742, Sa1808, Sa1896, Sa2320, and Sa1987 were used as potential indicator strains. For *P. aeruginosa*, Pa1315, Pa1400, Pa1414, Pa1499, Pa1641, Pa1651, Pa1786, Pa2302, Pa2371, K, O5(R18), O(pJB10), O1(pLM2), and O were used as potential indicator strains. For *K. pneumoniae*, Kp1447, Kp1468, Kp1473, Kp1586, Kp1627, Kp1752, Kp1823, Kp1897, Kp2329, Kp2362, and Kp2385 were used as potential indicator strains.

### Selected potential indicator strains for Set II

Cleared blood culture samples infected with *E. coli* from Set II were plated with the following 25 strains: Ec1255, Ec1321, Ec1457, Ec1507, Ec1522, Ec1572, Ec1643, Ec1675, Ec1685, Ec1748, Ec1758, Ec1798, Ec1809, Ec1816, Ec1905, Ec1910, Ec1998, Ec1999, Ec2000, Ec2311, Ec2392, Ec2400, Ec2407, Ec2424, and YMC. Samples infected with *S. aureus* or *K. pneumoniae* were plated with all *S. aureus* or *K. pneumoniae* strains from Set I (see Table 1), respectively. Samples infected with *P. aeruginosa* were plated with the following strains: Pa1315, Pa1400, Pa1414, Pa1641, Pa1651, Pa1786, Pa2302, Pa2371, O5(R18), and K.

Spontaneous plaque production by Set II bacterial isolates was tested using the same indicator strains as listed for Set I except that some additional strains were verified based on results obtained with blood culture samples.

### Phage induction

For UV induction, bacteria were grown to 200 Klett units and collected by centrifugation for 10 min at 6,000 rpm using a Sorvall SS-34 rotor at 4°C. Bacteria were suspended in the same volume of M9-broth and transferred to a glass Petri dish. The bacterial suspension was irradiated for 42 sec at A_254_ followed by dark storage on ice for 1 h. Cells were collected by centrifugation for 5 min at 13,000 rpm using a Heraeus Biofuge at 22°C. Bacteria were suspended in 3 volumes of LB and the number of plaques was determined after additional two h incubation at 37°C.

For MitC induction experiments, cells were grown to 200 Klett units and induced with MitC at a final concentration of 5 µg/ml. Cells were incubated for 15 min at 37°C and the growth medium was then replaced with fresh LB. Plaques were determined after additional two h incubation at 37°C.

To test if antibiotics could induce phage production, the same procedure was used as in the previous paragraph with the following modifications. Three different antibiotic concentrations were tested depending on the antibiotic used and bacterial strain employed. For *E. coli* strains, 1, 10, and 30 µg/ml final concentrations of tobramycin (tomycin, Orion Pharma) and 0.03, 0.3, and 3 µg/ml of ciprofloxacin (Bayer) were used. For *S. aureus* strains, 1, 10, 20 µg/ml final concentrations of tobramycin and 1, 10, 30 µg/ml of ciprofloxacin were used. Viable counts of cell suspensions were determined to evaluate the antibacterial activity of UV, MitC or antibiotic treatment.

### Phage purification

Phage stocks were obtained as follows. Soft agar from semi-confluent plates was collected and mixed with LB at 3 ml per plate, and incubated for 2 h at 37°C with aeration. Debris was removed by centrifugation for 20 min at 8,000 rpm using a Sorvall SS-34 rotor at 5°C. Phage particles were concentrated with 10% polyethylene glycol 6000 and 0.5 M NaCl and were purified twice by rate zonal centrifugation in a linear 5–20% (w/v) sucrose gradient prepared in 20 mM Tris, pH 7.2, 1 mM MgCl_2_, and 0.2 mM CaCl_2_ [22]. Protein composition of purified virus preparations was determined by SDS-PAGE [23].

### Electron microscopy

Purified virus specimens were applied to carbon-coated grids and were stained with 1% (w/v) potassium phosphotungstate (pH 6.5). Micrographs were obtained with a JEOL 1200 EX electron microscope (Institute of Biotechnology, University of Helsinki) operated at 60 kV.

## Supporting Information

Table S1 Blood culture samples and original bacterial isolates collected from the Helsinki University Hospital laboratory
(0.12 MB DOC)

Table S2 Characterized phage isolates
(0.07 MB DOC)
