# Supplementary material for: Bacteria, Phages and Septicemia
Source: PLoS One. 2007 Nov 7;2(11):e1145. doi: 10.1371/journal.pone.0001145 (PMC2190619; doi:10.1371/journal.pone.0001145)
Supplement: Table S1 — Blood culture samples and original bacterial isolates collected from the Helsinki University Hospital laboratory (0.12 MB DOC) [file pone.0001145.s001.doc]

**Table S1.**  Blood culture samples and original bacterial isolates collected from the Helsinki University Hospital laboratory.

### Set I

## *E. coli* n = 90 (90)

| Blood culture sample | Original  bacterial isolate | Blood culture sample | Original bacterial isolate | Blood culture sample | Original bacterial isolate |
| --- | --- | --- | --- | --- | --- |
| 05vv1255a | Ec1255a | 05vv1660 | Ec1660 ^UV^ | 05vv1891a | Ec1891a |
| 05vv1276a | Ec1276a | 05vv1661 | Ec1661 ^M^ | 05vv1899n | Ec1899n ^M^ |
| 05vv1309a | Ec1309a | 05vv1671 | Ec1671 | 05vv1901a | Ec1901a ^M^ |
| 05vv1319a | Ec1319a ^S, M, UV^ | 05vv1673 | Ec1673 ^M^ | 05vv1905n | Ec1905n |
| 05vv1321n | Ec1321n ^S, M, UV^ | 05vv1675 | Ec1675 | 05vv1910a | Ec1910a |
| 05vv1325n | Ec1325n | 05vv1685 | Ec1685 ^M^ | 05vv1971n | Ec1971n ^M^ |
| 05vv1338a | Ec1338a ^UV^ | 05vv1691 | Ec1691 | 05vv1976n | Ec1976n |
| 05vv1342a | Ec1342a ^M, UV^ | 05vv1706 | Ec1706 ^S, M, UV^ | 05vv1977n | Ec1977n ^M, UV^ |
| 05vv1377a | Ec1377a ^UV^ | 05vv1711 | Ec1711 | 05vv1979a | Ec1979a ^S, M, UV^ |
| 05vv1387a | Ec1387a ^S, M, UV^ | 05vv1725a | Ec1725a ^M, UV^ | 05vv1992a | Ec1992a ^S, M, UV^ |
| 05vv1457a | Ec1457a ^UV^ | 05vv1726a | Ec1726a ^S, M, UV^ | 05vv1994a | Ec1994a ^S, M, UV^ |
| 05vv1462a | Ec1462a | 05vv1731a | Ec1731a ^S^ | 05vv1998a | Ec1998a ^M, UV^ |
| 05vv1484a | Ec1484a ^M, UV^ | 05vv1738a | Ec1738a | 05vv1999a | Ec1999a ^S, M, UV^ |
| 05vv1492a | Ec1492a | 05vv1747n | Ec1747n ^S, M, UV^ | 05vv2000a | Ec2000a ^S, UV^ |
| 05vv1507a | Ec1507a | 05vv1748a | Ec1748a | 05vv2311 | Ec2311 ^M^ |
| 05vv1513 | Ec1513 | 05vv1758a | Ec1758a | 05vv2327 | Ec2327 ^UV^ |
| 05vv1520a | Ec1520a ^UV^ | 05vv1778a | Ec1778a | 05vv2334 | Ec2334 ^UV^ |
| 05vv1522a | Ec1522a ^S, M, UV^ | 05vv1779a | Ec1779a | 05vv2336n | Ec2336n ^UV^ |
| 05vv1537n | Ec1537n | 05vv1781a | Ec1781a | 05vv2341a | Ec2341a |
| 05vv1557a | Ec1557a ^UV^ | 05vv1782a | Ec1782a | 05vv2364a | Ec2364a ^S, M^ |
| 05vv1558a | Ec1558a ^S, M, UV^ | 05vv1790 | Ec1790 ^S, M, UV^ | 05vv2367n | Ec2367n ^M, UV^ |
| 05vv1564a | Ec1564a ^S, UV^ | 05vv1792 | Ec1792 ^S, M^ | 05vv2388 | Ec2388 ^S, M, UV^ |
| 05vv1572a | Ec1572a ^M, UV^ | 05vv1798 | Ec1798 | 05vv2392a | Ec2392a ^M^ |
| 05vv1636 | Ec1636 ^UV^ | 05vv1809a | Ec1809a ^S, M, UV^ | 05vv2400n | Ec2400n |
| 05vv1639 | Ec1639 | 05vv1816 | Ec1816 | 05vv2407 | Ec2407 |
| 05vv1643 | Ec1643 | 05vv1826 | Ec1826 ^S^ | 05vv2408 | Ec2408 ^M, UV^ |
| 05vv1653 | Ec1653 ^M, UV^ | **05vv1871a** | Ec1871a ^S, M, UV^ | 05vv2414a | Ec2414a ^M, UV^ |
| 05vv1654 | Ec1654 ^UV^ | 05vv1874n | Ec1874n ^S, M, UV^ | 05vv2415a | Ec2415a ^UV^ |
| 05vv1657 | Ec1657 | 05vv1878a | Ec1878a ^M, UV^ | 05vv2418a | Ec2418a ^S, M, UV^ |
| 05vv1658 | Ec1658 ^S, M, UV^ | 05vv1889a | Ec1889a ^UV^ | 05vv2424a | Ec2424a |

***S. aureus*** n = 26 (26)

| Blood culture sample | Original bacterial isolate | Blood culture sample | Original bacterial isolate | Blood culture sample | Original bacterial isolate |
| --- | --- | --- | --- | --- | --- |
| 05vv1252a | Sa1252a ^S, M, UV^ | 05vv1582a | Sa1582a ^S, M, UV^ | 05vv1904a | Sa1904a |
| 05vv1263a | Sa1263a ^S^ | 05vv1631 | Sa1631 | 05vv1912a | Sa1912a ^UV^ |
| 05vv1277a | Sa1277a | 05vv1742n | Sa1742n ^M^ | 05vv1970n | Sa1970n |
| 05vv1303a | Sa1303a | 05vv1784a | Sa1784a | 05vv1987a | Sa1987a ^S, M, UV^ |
| 05vv1433 | Sa1433 ^M^ | 05vv1795 | Sa1795 | 05vv2012a | Sa2012a ^M, UV^ |
| 05vv1439a | Sa1439a ^M^ | 05vv1808 | Sa1808 | 05vv2289a | Sa2289a ^S, M, UV^ |
| 05vv1465a | Sa1465a | 05vv1818 | Sa1818 ^M^ | 05vv2320 | Sa2320 ^M^ |
| 05vv1469a | Sa1469a | 05vv1865n | Sa1865n ^M^ | 05vv2345a | Sa2345a ^S, M^ |
| 05vv1554 | Sa1554 ^UV^ | 05vv1896a | Sa1896a ^S, M, UV^ |  |  |

***P. aeruginosa*** n = 16 (16)

| Blood culture sample | Original  bacterial isolate | Blood culture sample | Original bacterial isolate | Blood culture sample | Original bacterial isolate |
| --- | --- | --- | --- | --- | --- |
| 05vv1315a | Pa1315a ^S, M, UV^ | 05vv1669 | Pa1669 * ^S, M, UV^ | 05vv2026 | Pa2026 * ^S, M^ |
| 05vv1400a | Pa1400a ^S, M, UV^ | 05vv1701 | Pa1701 * ^S, M, UV^ | 05vv2302 | Pa2302 ^S, M, UV^ |
| 05vv1414a | Pa1414a ^S, M, UV^ | 05vv1745a | Pa1745a * ^S, M, UV^ | 05vv2322 | Pa2322 * ^S, M, UV^ |
| 05vv1499a | Pa1499a * ^S, M, UV^ | 05vv1786a | Pa1786a ^S, M, UV^ | 05vv2371a | Pa2371a ^S, M, UV^ |
| 05vv1641 | Pa1641 ^S, M, UV^ | 05vv1973a | Pa1973a * ^S, M, UV^ |  |  |
| 05vv1651 | Pa1651 ^S, M, UV^ | 05vv2008a | Pa2008a * ^S, M, UV^ |  |  |

***K. pneumoniae*** n = 17 (18)

| Blood culture sample | Original  bacterial isolate | Blood culture sample | Original bacterial isolate | Blood culture sample | Original bacterial isolate |
| --- | --- | --- | --- | --- | --- |
| 05vv1447 | Kp1447 | 05vv1627a | Kp1627a | 05vv1897a | Kp1897a ^S, M, UV^ |
| 05vv1468n | Kp1468n | 05vv1718 | Kp1718 | 05vv2293a | Kp2293a |
| 05vv1473a | Kp1473a | 05vv1752n | Kp1752n | 05vv2329 | Kp2329 |
| 05vv1482a | Kp1482a | 05vv1771 | Kp1771 | 05vv2343a | Kp2343a ^S, M, UV^ |
| 05vv1586n | Kp1586n | 05vv1823 | Kp1823 | 05vv2362a | Kp2362a |
| 05vv1622 | Kp1622 | **05vv1871a** | Kp1871a | 05vv2385a | Kp2385a |

### Set II

## *E. coli* n = 14 (14)

| Blood culture sample | Original  bacterial isolate | Blood culture sample | Original bacterial isolate | Blood culture sample | Original bacterial isolate |
| --- | --- | --- | --- | --- | --- |
| 06vv2955 | Ec06_2955 | 06vv3029a | Ec06_3029a | 06VT146a | Ec06_VT146a ^S^ |
| 06vv2961a | Ec06_2961a | 06vv3107n | Ec06_3107n | 06vv3183n | Ec06_3183n ^S^ |
| 06vv2974n | Ec06_2974n ^S^ | 06vv3108a | Ec06_3108a | 06vv3242 | Ec06_3242 |
| 06vv2987a | Ec06_2987a ^S^ | 06vv3119n | Ec06_3119n ^S^ | 06vv3247 | Ec06_3247 |
| 06vv2988a | Ec06_2988a | 06vv3140a | Ec06_3140a |  |  |

***S. aureus*** n = 12 (12)

| Blood culture sample | Original  bacterial isolate | Blood culture sample | Original bacterial isolate | Blood culture sample | Original bacterial isolate |
| --- | --- | --- | --- | --- | --- |
| 06VT26 | Sa06_VT26 | 06vv3118a | Sa06_3118a ^S^ | 06vv3184n | Sa06_3184n |
| 06vv2969a | Sa06_2969a ^S^ | 06vv3106a | Sa06_3106a | 06vv3189n | Sa06_3189n ^S^ |
| 06vv2986a | Sa06_2986a ^S^ | 06vv3133n | Sa06_3133n ^S^ | 06vv3193n | Sa06_3193n |
| 06vv3057a | Sa06_3057a | 06VT148a | Sa06_VT148a | 06vv3244 | Sa06_3244 ^S^ |

***P. aeruginosa*** n = 3 (3)

| Blood culture sample | Original  bacterial isolate | Blood culture sample | Original bacterial isolate | Blood culture sample | Original bacterial isolate |
| --- | --- | --- | --- | --- | --- |
| 06vv2991a | Pa06_2991a | 06vv3141a | Pa06_3141a | 06vv3241 | Pa06_3241 |
|  |  |  |  |  |  |

***K. pneumoniae*** n = 1 (1)

| Blood culture sample | Original  bacterial isolate | Blood culture sample | Original bacterial isolate | Blood culture sample | Original bacterial isolate |
| --- | --- | --- | --- | --- | --- |
| 06VT145a | Kp06_VT145a |  |  |  |  |
|  |  |  |  |  |  |

The blood sample containing two bacterial species is indicated in bold. * strains that produced phages detectable on their own lawns; S, strains that produced phages spontaneously; M, strains that produced phages upon MitC induction; UV, strains that produced phages upon UV induction; n, number of blood culture samples (number of original bacterial isolates is given in parentheses).
