# Supplementary material for: Bacteria, Phages and Septicemia
Source: PLoS One. 2007 Nov 7;2(11):e1145. doi: 10.1371/journal.pone.0001145 (PMC2190619; doi:10.1371/journal.pone.0001145)
Supplement: Table S2 — Characterized phage isolates (0.07 MB DOC) [file pone.0001145.s002.doc]

**Table S2.**  Characterized phage isolates. (A) Set I and (B) Set II.

## A

| Phage^a^ | Origin | Host strain^b^ | Titer of virus stock (PFU/ml) | Morphology^c^ |
| --- | --- | --- | --- | --- |
| ***E. coli* phages** |  |  |  |  |
| Phi05_1558 | M, S, UV | Ec1522 | 2.5x10^8^ | myo |
| Phi05_1658 | M, S, UV | YMC | 6.4x10^8^ | sipho |
| Phi05_1706 | M, S, UV | YMC | 8.0x10^8^ | sipho |
| Phi05_1999 | M, S, UV | Ec2424 | 5.7x10^10^ | podo |
| Phi05_1387 | UV, S, M | Ec1457 | 7.1x10^9^ | myo |
| Phi05_2388 | UV, S, M | Ec1643 | 2.4x10^10^ | podo |
| Phi05_1387 | B | Ec1457 | 3.8x10^9^ | myo |
| Phi05_1558 | B | Ec1522 | 2.1x10^8^ | myo |
| Phi05_1999 | B | Ec2424 | 8.3x10^10^ | podo |
| Phi05_2388 | B | Ec1643 | 8.1x10^9^ | podo |
| ***S. aureus* phages** |  |  |  |  |
| Phi05_1433 | M | Sa1469 | 7.3x10^10^ | sipho |
| Phi05_2289 | M, S, UV | Sa1303 | 1.9x10^11^ | sipho |
| Phi05_1554 | UV | Sa1554 | 1.7x10^10^ | sipho |
| ***P. aeruginosa* phages** |  |  |  |  |
| Phi05_1315 | M, S, UV | Pa1400 | 5.0x10^10^ | sipho |
| Phi05_1400 | M, S, UV | Pa1414 | 4.5x10^7^ | sipho |
| Phi05_1786 | M, S, UV | PAO5(R18) | 1.1x10^11^ | filament |
| Phi05_2302 | UV, S, M | PAO5(R18) | 1.5x10^11^ | filament |
| Phi05_1669 | S | Pa1400 | 6.0x10^10^ | sipho |
| Phi05_1745 | S | Pa1400 | 8.2x10^10^ | sipho |
| Phi05_1973 | S | Pa1651 | 4.9x10^10^ | sipho |
| Phi05_2322 | S | Pa1400 | 7.1x10^10^ | sipho |
| Phi05_1315 | B | Pa1400 | 4.5x10^10^ | sipho |
| Phi05_1400 | B | Pa1414 | 2.0x10^10^ | sipho |
| ***K. pneumoniae* phages** |  |  |  |  |
| Phi05_2343 | B | Kp1752 | 2.3x10^5^ | myo |

**B**

| Phage^a^ | Origin | Host strain^b^ | Titer of virus stock (PFU/ml) | Morphology^c^ |
| --- | --- | --- | --- | --- |
| ***E. coli* phages** |  |  |  |  |
| Phi06_2974 | S | Ec1457 | 6.1x10^10^ | myo |
| Phi06_2987 | S | YMC | 2.7x10^9^ | sipho |
| Phi06_3242 | S | Ec1522 | 5x10^10^ | podo |
| Phi06_2974 | B | Ec1457 | 7x10^8^ | myo |
| Phi06_2987 | B | YMC | 7x10^9^ | sipho |
| Phi06_3242 | B | Ec1522 | 6x10^10^ | podo |
| ***S. aureus* phages** |  |  |  |  |
| Phi06_2986 | S | Sa1912 | 4.4x10^11^ | sipho |
| Phi06_3244 | S | Sa1912 | 1.7x10^11^ | sipho |
| Phi06_2986 | B | Sa1912 | 9x10^10^ | sipho |
| Phi06_3106 | B | Sa1912 | 5.4x10^10^ | sipho |
| Phi06_3244 | B | Sa1263 | 1.7x10^11^ | sipho |
| ***K. pneumoniae* phages** |  |  |  |  |
| Phi06_VT145 | B | Kp1752 | 5x10^10^ | myo |

^a^ Phage numbers indicate the bacterial strain or blood culture sample from where the phage was isolated.

^b^ The host strain used was the one that gave the highest plating efficiency out of 10 or more selected potential indicator strains.

^c^ Phage morphology was assigned based on electron microscopy analysis.

M, MitC induction; UV, UV induction; S, spontaneous induction; B, blood culture sample.
